# Cardiovascular responses to high‐intensity stair climbing in individuals with coronary artery disease

**DOI:** 10.14814/phy2.15308

**Published:** 2022-05-19

**Authors:** Sydney E. Valentino, Emily C. Dunford, Jonathan Dubberley, Eva M. Lonn, Martin J. Gibala, Stuart M. Phillips, Maureen J. MacDonald

**Affiliations:** ^1^ 3710 Department of Kinesiology McMaster University Hamilton Ontario Canada; ^2^ 3708 Hamilton Health Sciences Hamilton Ontario Canada; ^3^ 3708 Population Health Research Institute Hamilton Ontario Canada; ^4^ 3710 Department of Medicine McMaster University Hamilton Ontario Canada

**Keywords:** cardiac function, cardiac rehabilitation, flow‐mediated dilation, HIIT, stair climbing

## Abstract

Exercise‐based cardiac rehabilitation leads to improvements in cardiovascular function in individuals with coronary artery disease. The cardiac effects of coronary artery disease (CAD) can be quantified using clinical echocardiographic measures, such as ejection fraction (EF). Measures of cardiovascular function typically only used in research settings can provide additional information and maybe more sensitive indices to assess changes after exercise‐based cardiac rehabilitation. These additional measures include endothelial function (measured by flow‐mediated dilation), left ventricular twist, myocardial performance index, and global longitudinal strain. To investigate the cardiovascular response to 12 week of either traditional moderate‐intensity (TRAD) or stair climbing‐based high‐intensity interval (STAIR) exercise‐based cardiac rehabilitation using both clinical and additional measures of cardiovascular function in individuals with CAD. Measurements were made at baseline (BL) and after supervised (4wk) and unsupervised (12 week) of training. This study was registered as a clinical trial at clinicaltrials.gov (NCT03235674). Participants were randomized into either TRAD (*n* = 9, 8M/1F) and STAIR (*n* = 9, 8M/1F). There was a training‐associated increase in one component of left ventricular twist: Cardiac apical rotation (TRAD: BL: 5.6 ± 3.3º, 4 week: 8.0 ± 3.9º, 12 week: 6.2 ± 5.1º and STAIR: BL: 5.1 ± 3.6º, 4 week: 7.4 ± 3.9º, 12 week: 7.8 ± 2.8º, *p* (time) = 0.03, η^2^ = 0.20; main effect) and post‐hoc analysis revealed a difference between BL and 4 week (*p *= 0.02). There were no changes in any other clinical or additional measures of cardiovascular function. The small increase in cardiac apical rotation observed after 4 weeks of training may indicate an early change in cardiac function. A larger overall training stimulus may be needed to elicit other cardiovascular function changes.

## Graphical abstract

No changes in endothelial function or clinical cardiac function measures after either traditional or stair climbing‐based high‐intensity interval training. After 4 weeks of exercise training, we observed an increase in cardiac apical rotation in individuals with coronary artery disease, which may indicate an early change in cardiac function. Other cardiovascular structural and functional adaptations may be dependent upon both duration of training and baseline fitness status.

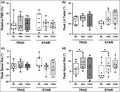

## 1 INTRODUCTION

The extent of endothelial dysfunction, atherosclerosis, ventricular remodeling, and the associated functional changes that occur with a myocardial infarction depends on the location and severity of coronary artery disease (CAD) and the characteristics of the infarct (Eržen et al., 2007; Pfeffer & Braunwald, 1990). Clinical echocardiography measures, including ejection fraction and cardiac volumes, are an essential part of evaluating the diagnosis and prognosis of CAD (Ehsani et al., 1978; Giannuzzi et al., 2003; Haykowsky et al., 2007; Pastore et al., 2021). However, additional ultrasound‐based cardiac and endothelial function measures, such as speckle tracking echocardiography and brachial artery flow‐mediated dilation (FMD), have been extensively studied in research settings. These additional parameters may provide enhanced prognosis and monitoring information about the cardiac and vascular function (Awadalla et al., 2017; Kou et al., 2011; Thijssen et al., 2011) and facilitate the detection of more subtle changes in cardiovascular function after interventions such as exercise‐based cardiac rehabilitation (Awadalla et al., 2017; Kou et al., 2011). Brachial artery FMD has proven effective as a non‐invasive and independent predictor of cardiac events (Green et al., 2011; Al et al., 2000) and a surrogate for coronary artery function (Anderson et al., 1995; Takase et al., 1998). Speckle‐tracking echocardiography outcomes, including left ventricular twist, myocardial performance index, and global longitudinal strain, have been validated for diagnosis and risk stratification in different cardiac diseases (Pastore et al., 2021). Together, these ultrasound‐derived measures provide a comprehensive understanding of resting cardiovascular function.

Engagement in exercise‐based cardiac rehabilitation leads to a lowered risk of myocardial event reoccurrence and re‐hospitalization in individuals with CAD (Giannuzzi et al., 2008). Exercise training is often prescribed as part of cardiac rehabilitation and has been demonstrated to improve blood flow to the myocardium and mitigate the progression of CAD (Lavie & Bennett, 2017; McKay et al., 1986). Exercise‐based cardiac rehabilitation typically focuses on moderate‐intensity exercise training, also known as traditional exercise‐based cardiac rehabilitation (TRAD), as the accepted exercise training method (Fletcher et al., 2013; Thomas et al., 2007). However, a systematic review and meta‐analysis showed that high‐intensity interval training (HIIT), in comparison to moderate‐intensity continuous training, improved peak oxygen uptake for individuals post‐myocardial infarction (Qin et al., 2021). Stair climbing is an alternative modality of exercise training that has been shown to lead to an improvement in arterial stiffness after 12 weeks in hypertensive post‐menopausal women (Wong et al., 2018). Stair climbing‐based HIIT (STAIR), is a minimal‐equipment, low‐cost, and time‐efficient option that addresses commonly cited barriers to exercise‐based cardiac rehabilitation including access to facilities, gym fees, and lack of time (Daly et al., 2002). This exercise training modality leads to improvements in cardiorespiratory fitness in individuals with CAD and was well‐tolerated in individuals with type 2 diabetes mellitus (Dunford et al., 2021; Godkin et al., 2018). However, there is limited research that has comprehensively examined both cardiac and vascular adaptations after the completion of exercise‐based cardiac rehabilitation.

We investigated the effect of TRAD compared to STAIR training on cardiovascular function using both clinical and additional measures. Informed by recent evidence regarding the effect of moderate‐intensity exercise training compared to a non‐exercise control (Kou et al., 2011), we hypothesized that brachial artery FMD, left ventricular twist, and myocardial performance index would improve after HIIT to the same extent as TRAD. Additionally, in alignment with the previously observed time course for changes in clinical measures of cardiac function (Ehsani et al., 1978; Giannuzzi et al., 2003; Haykowsky et al., 2007), we hypothesized there would be no change in global longitudinal strain, ejection fraction or other clinical indices of cardiac function in both TRAD and HIIT.

## 2 METHODS

### 2.1 Participants

Participants were recruited from the Cardiac Health and Rehabilitation Centre at the Hamilton General Hospital. Participants needed to be registered for cardiac rehabilitation and had a history of myocardial infarction, coronary artery bypass graft, and/or percutaneous coronary intervention. The ineligibility criteria included any patients that had previously participated in rehabilitation for the same cardiac event, had a non‐cardiac surgical procedure <2 months prior to recruitment, a pacemaker or atrial fibrillation, documented peak orifice area valve stenosis, symptomatic peripheral arterial disease that limits exercise capacity, unstable angina, uncontrolled hypertension (blood pressure >180/100 mmHg), documented chronic obstructive pulmonary disease (FEV1 < 60% and/or FVC < 60%) and any musculoskeletal abnormality that would limit exercise participation. The study protocols were approved by the Hamilton Integrated Research Ethics Board (HIREB #3301) and conform to the *Declaration of Helsinki* concerning the use of human subjects as research participants. Written and verbal informed consent were obtained from all subjects. This non‐inferiority, repeated measures study was pre‐registered on clinicaltrials.gov (NCT03235674) and the primary outcome was endothelial function.

### 2.2 Study design and protocol

A detailed protocol has previously been published (Dunford et al., 2021). Our study included two phases to mimic the timeline of exercise‐based cardiac rehabilitation at the Cardiac Health and Rehabilitation Centre in Hamilton. The initial phase involved supervised exercise training for six individuals, supervised exercise sessions (approximately 4 week) and the second phase of unsupervised exercise performed at the participants’ choice of either at‐home or a community‐based facility for an additional 8 weeks (total of 12 week). During the unsupervised training, participants were encouraged to train at a frequency of three times per week. All supervised sessions had a registered kinesiologist present. All cardiovascular assessments were conducted at McMaster University at baseline (BL), following phase one (4 week), and phase two (12 week). A randomization scheme was generated by one study investigator using an online website (https://www.randomization.com) to randomize participants to one of two treatment groups, either TRAD or STAIR, and a second study investigator revealed the group allocation to each participant after they had provided verbal and written consent. Prior to enrollment, cardiopulmonary exercise tests were conducted at the Hamilton General Hospital under physician supervision for measurement of peak cardiorespiratory fitness (V̇O_2peak_), and peak heart rate (HR_peak_) (Dunford et al., 2021

### 2.3 Exercise interventions

During each training session, the TRAD group participants were advised to accumulate at least 30 min of moderate‐intensity exercise using a combination of self‐paced walking and/or a combination of equipment including a stationary cycle ergometer and treadmill. They also performed a 10‐min warm‐up and 5‐min cool‐down. Regardless of the modality, the workload was designed to elicit 60–80% of the individual heart rate reserve (HRR) determined from the pre‐training cardiopulmonary exercise tests and with an intensity goal of 11–13 on Borg's ratings of perceived exertion (RPE) 6–20 scale (Borg et al., 1987).

During each training session, the STAIR group completed a HIIT exercise session modeled after a previous study involving young women (Allison et al., 2017). For this protocol, the participants completed self‐paced walking for a duration of 10 min for the warm‐up and 5 min for the cool down. Each session consisted of three ascents of a single flight of stairs (12 steps), repeated six times at a self‐selected “vigorous” pace, separated by 90‐s periods of self‐paced walking on flat ground. The participants were instructed to “climb up and down the stairs one step at a time. Ascend at a pace that you find challenging, and descend at a pace you find comfortable, such that you feel you can safely manage the three bouts of stair climbing. Use the railings for support if you wish.” Each bout thus involved ascending and descending a total of 72 steps. Participants were instructed to aim for either 14 or 15 out of 20 on the 6–20 RPE scale, and their achieved RPE was recorded at the end of each bout of both high‐intensity (ascending stairs) and low‐intensity (descending stairs) intervals. For STAIR, percent HRR (%HRR) was retrospectively calculated for comparing to TRAD but was not used for exercise prescription purposes.

As previously described, adherence was defined as the percentage of sessions completed. During the supervised period of exercise based cardiac rehabilitation (weeks 0–4) exercise, six sessions were defined as 100% completion. During the unsupervised period (weeks 4–12), 12 sessions of exercise training were defined as 100% completion (Dunford et al., 2021).

## 3 MEASUREMENTS

### 3.1 Cardiopulmonary exercise test

Participants completed the test on either a stationary bicycle or treadmill during a medically supervised cardiopulmonary exercise test using a ramp protocol, as previously reported (Dunford et al., 2021). The modality of exercise during the cardiopulmonary exercise test was consistent for each participant across all time points.

### 3.2 Additional measures and study design

In advance of each testing session, participants were asked to refrain from exercise and alcohol consumption for 24 h, avoid caffeine ingestion for 10 h and to fast overnight (at least 8 h). Participants were typically prescribed vasoactive medication (i.e., nitroglycerin) to use on an as‐needed basis; hence they verbally confirmed that they did not need to use this medication in the 24 h before testing start time. All other prescribed medications and vitamins were consumed as usual. Additional testing was conducted at the Vascular Dynamics Laboratory at McMaster University at the same time of day in the morning. Prior to any assessments, participants rested in the supine position for 10 min to ensure a steady‐state baseline was achieved for all cardiovascular parameters. Each testing session visit began with physiological measurements, including HR, blood pressure (BP), standing height, and body mass. Three resting supine BP measures were assessed via brachial artery oscillometry (Dinamap V100; GE Healthcare). A fourth value was obtained if the systolic BP differed by greater than or equal to 10 mmHg, and the closest three measurements were averaged. Vascular and cardiac ultrasound assessments were completed using a commercial ultrasound unit (Vivid Q; GE Medical Systems, Horten, Norway) with simultaneous electrocardiogram (ECG Vivid Q, GE Medical Systems, Horten, Norway) with the participant resting in a supine position.

### 3.3 Flow‐mediated dilation (FMD)

Brachial artery endothelial‐dependent function was measured using the non‐invasive FMD test, according to the current guidelines (Thijssen et al., 2011). A BP cuff was positioned on the forearm, immediately distal to the antecubital fossa, and a 30‐s baseline image was acquired. The cuff was inflated using a rapid cuff inflator (AG101 cuff inflator air source, Hokanson, Washington, USA), at the highest pressure of either 200 mmHg or a minimum of 50 mmHg above systolic blood pressure for 5 min to occlude blood flow to the distal vascular bed, as per the guidelines (Thijssen et al., 2019). Longitudinal images of the brachial artery were obtained using a 12‐MHz linear array probe proximal to the antecubital fossa. All images were collected in duplex mode to obtain both brightness mode images and pulse wave velocity profiles, with an insonation angle of 68° (Pyke et al., 2008). A continuous recording of reactive hyperemia images began 5 s before cuff deflation and continuously for three minutes afterward.

Brachial artery ultrasound images were stored in Digital Imaging and Communications in Medicine (DICOM) format. For the analysis of the FMD test, end‐diastolic frames were extracted from each heart cycle and compiled into a new DICOM file (Sante DICOM Editor, v. 3.1.20, Santesoft, Athens, Greece), which was analyzed for arterial diameters using semiautomated edge tracking software [Artery Measurement System (AMS) II, version 1.141, Gothenburg, Sweden] (Wendelhag et al., 1997). All arterial analysis was completed with the researcher blinded to the testing visit (BL, 4 week, 12 week). All equations for calculations of resting and peak reactive hyperemia diameter, absolute and relative FMD, mean blood velocity (MBV), peak reactive hyperemia blood flow (peak RHBF), blood flow (BF), shear rate (SR), MBV averaged to peak reactive hyperemia diameter (MBV to peak), SR area under the curve to peak reactive hyperemia diameter (SR AUC to peak), and time to peak reactive hyperemia diameter (time to peak), are published in Shenouda et al. (2018), as per laboratory protocol.

### 3.4 Cardiac assessments

All cardiac measures were assessed via non‐invasive ultrasound imaging of the heart. The ultrasound operator obtained the ultrasound measures from the left side of the participants thoracic cage with the ultrasound probe placed between the ribs for the sharpest acoustic window. Short video segments of at least five heart cycles were recorded at an approximate depth of 13 cm and at a frame rate of 30–50 fps, using a 1.5–3.6 MHz sector phased‐array probe. The views obtained included the parasternal short axis (PSAX) view at the base of the left ventricle obtained at the level of both the mitral valve (MV) and the apex (AP), a parasternal long axis (PLAX) view, an apical four‐chamber (A4C) view in 2D and pulse wave (PW) mode, and an apical five‐chamber (A5C) view in PW mode. Data was stored offline and later analyzed using commercially available software (EchoPAC 110.0.2; GE Medical Systems, Horten, Norway). All measurements were conducted in triplicate, using the clearest three consecutive heart cycles of each ultrasound cineloop when possible, then averaged. All cardiac analysis was completed with the researcher blinded to the testing visit (BL, 4 week, 12 week).

### 3.5 Left ventricular twist

**FIGURE 1:**
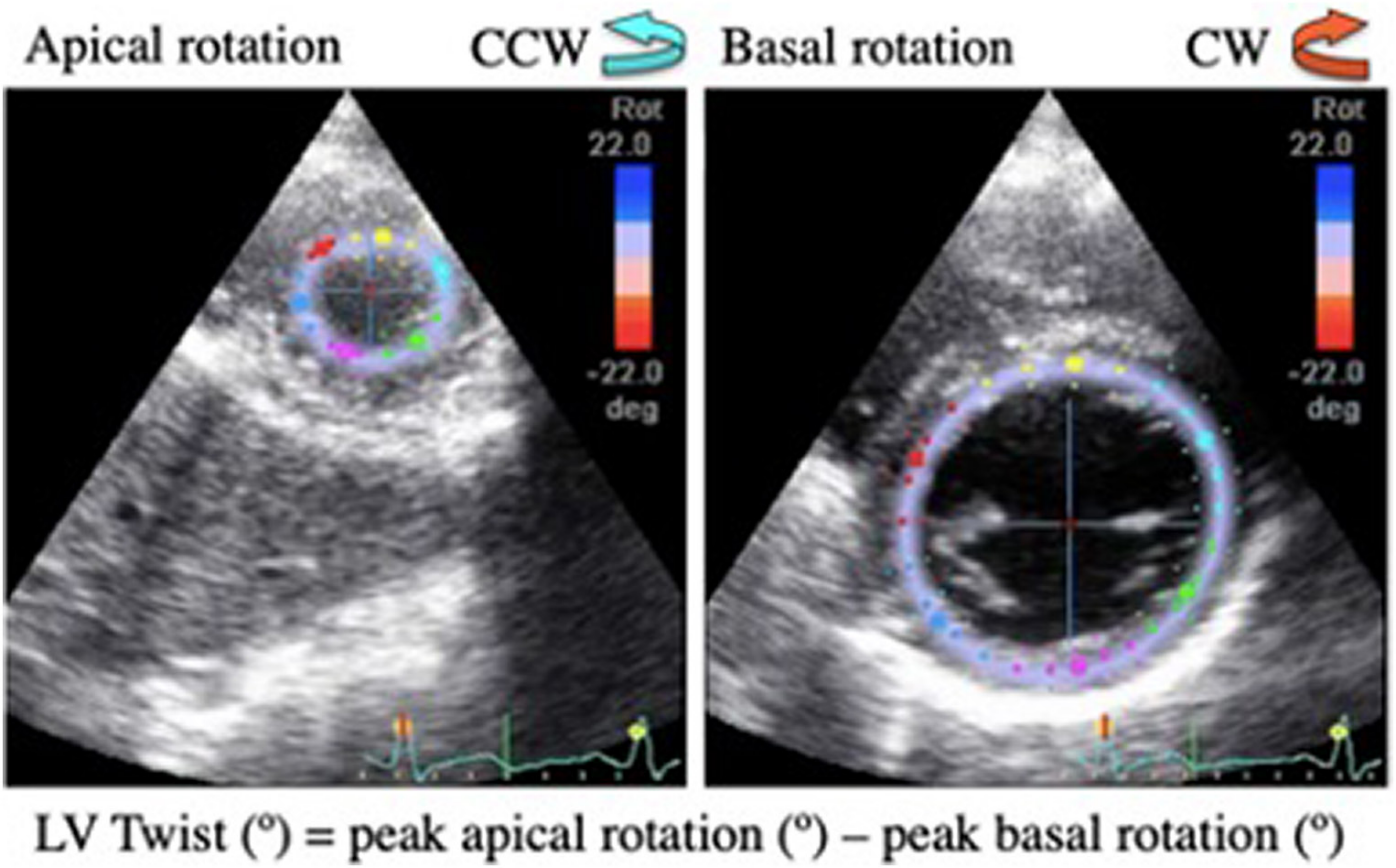
Speckle‐tracking echocardiography analysis of PSAX‐AP and PSAX‐MV images and the subsequent calculation required to obtain LV twist

Basal and apical cardiac rotation were analyzed via two‐dimensional speckle tracking echocardiography (Q‐analysis, Echo‐PAC PC, Version 110.0.2; GE Medical Systems, Horten, Norway). The overall rotation of the left ventricular, termed left ventricular twist, was calculated as the instantaneous difference between the peak apical and peak basal rotation (Dong et al., 1999) (see equation in Figure 1). The inner myocardial wall borders were manually traced for three heart cycles of the PSAX‐MV and PSAX‐AP views to obtain basal rotation and apical rotation, respectively (Figure 1). Subsequently, using the standard analysis package associated with this measure (Q‐analysis), an automated quality analysis of the ability to track the six different wall segments across the heart cycles was performed. If the speckle‐tracking was not accurate for 85% of the heart cycle, an alternate speckle location was chosen. Often an alternate heart cycle was chosen until the best option was determined, and then the final traces were reviewed for quality. After this image analysis quality check was complete, drift compensation was applied to bring the traces through the x‐axis by assuming constant estimation error over the cardiac cycle to reduce erroneous baseline shift. The same rater performed these practices in accordance with current guidelines (Lang et al., 2015). The circumferential and radial parameters generated from this analysis were then exported to excel files for further trace analysis with the 2D Strain Analysis Tool (Stuttgart, Germany), which uses cubic spline interpolation for all the traces to 1200 points and calculates peak values for statistical analysis.

### 3.6 Myocardial performance index

**FIGURE 2:**
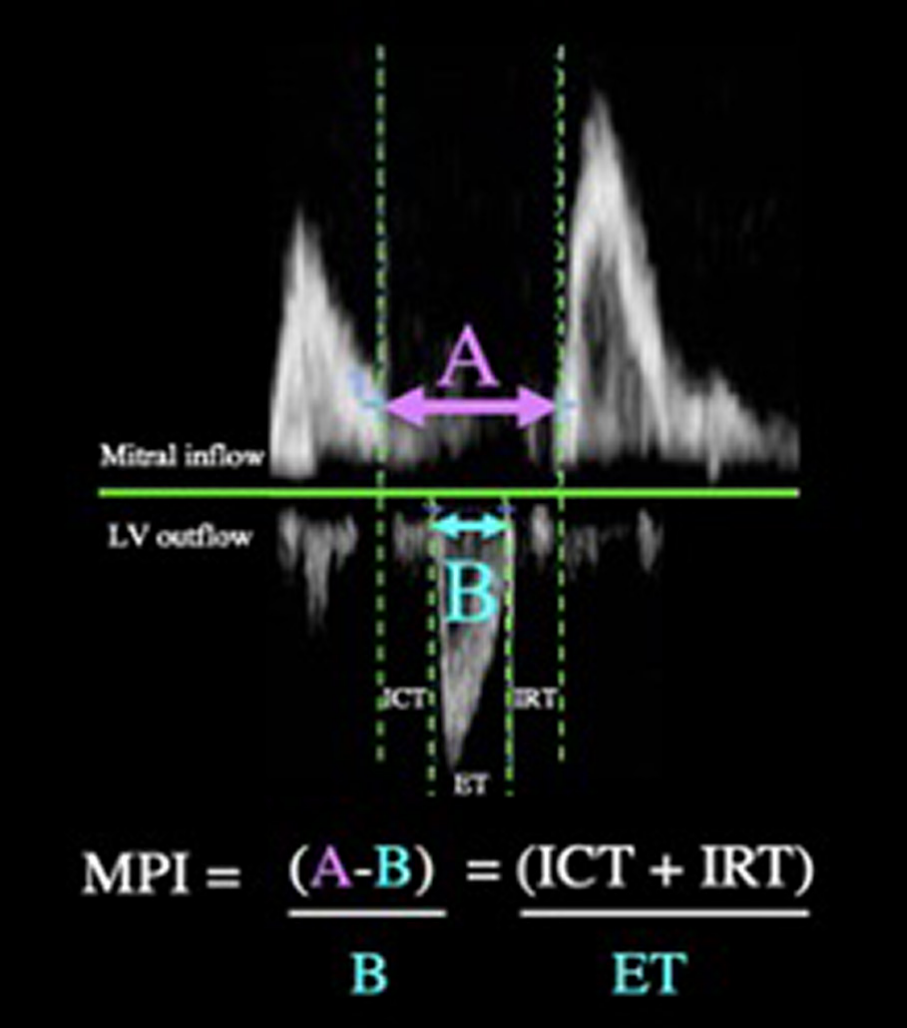
Representative image of MPI analysis from Doppler tracings of mitral inflow and LV outflow. A = time between filling periods; B = ejection time (ET). The sum of is isovolumic contraction time (ICT) and isovolumic relaxation time (IRT) can be obtained by the subtraction of B from A. Image is not to scale for the purpose of depicting the analysis graphically

We evaluated myocardial performance index from an average of three cardiac cycles using Doppler time intervals obtained from the left ventricular outflow (A5C‐PW mode) and the left ventricular inflow (A4C‐PW mode) measurements, as represented in Figure 2. The sum of isovolumic contraction time (ICT) and isovolumic relaxation time (IRT) was obtained by subtracting ejection time (ET) (Figure 2b) from the time interval between two mitral adjacent inflow periods (Figure 2a). Myocardial performance index then was determined as [(a − b)/b], where *a* is the time interval between two mitral inflow periods and *b* is ejection time (Qin et al., 2021).

### 3.7 Global longitudinal strain

Global longitudinal strain was obtained using the A4C view and tracing the left ventricular endocardium at the tissue‐blood interface without including the trabeculae. Using Q‐analysis, six segments of the left ventricle were tracked, including basal septal, mid septal, apical septal, apical lateral, mid lateral, and basal lateral segments. All tracking quality of all six segments were passed through an automatic checking system identical to that described above for left ventricular twist measures. Furthermore processing was completed using the 2DStrain tool, as described above.

### 3.8 Other cardiac measures

Clinical measures of cardiac structure and function were obtained from various echocardiographic views as follows: Cardiac output (CO) was measured using the left ventricular outflow tract velocity time integral to obtain stroke volume (SV), which was measured just below the aortic valve, and the ECG tracing to obtain HR, calculated as: (HR × SV). End‐diastolic volume (EDV) and end‐systolic volume (ESV) and were determined using the Simpson's monoplane method (Lang et al., 2015), and from these, SV was determined.

To measure diastolic filling of the left ventricle, measurements were completed using the left ventricular inflow from the A4C‐PW mode velocity tracing with the PW sample volume positioned at the tips of the mitral leaflets. The peaks of both the early active filling (E‐wave) and late passive filling (A‐wave) velocity waves were assessed, and the ratio was calculated (E/A ratio). Left ventricular mass is commonly represented as the thickness of the inner and outer left ventricular wall in diastole, measured using the PLAX view, and was calculated using the linear method shown in Equation 1, where 1.04 is the specific gravity of the myocardium (g/cm^3^), LVEDD is left ventricular end‐diastolic dimension (mm), IVS_d_ is the intraventricular septal thickness at end‐diastole (mm), PWT_d_ is posterior wall thickness at end‐diastole (mm) (Lang et al., 2015):

(1) $$\begin{array}{c} \text{LV mass}\left(g\right)=\\ 0.8\left\{1.04\left[\left({\left[\text{LVEDD}+{\text{IVS}}_{d}+{\text{PWT}}_{d}\right]}^{3}‐{\text{LVEDD}}^{3}\right)\right]\right\}+0.6 \end{array}$$

### 3.9 Statistical analysis

An *a priori* sample size calculation was computed using G*Power (Faul et al., 2007) (version 3.1.9.2; Windows) using data for the primary outcome variable of endothelial function. Previous data collected in our lab reported an improvement in endothelial function, measured by FMD, after 12 week of supervised HIIT on a stationary bicycle in individuals with CAD (Currie et al., 2013). We assumed a standard deviation of the change in relative FMD from baseline to follow‐up of 3.3% in each group. We then estimated that to detect a difference of 1.7% in the change of relative FMD from baseline to the end of the intervention, a minimum of 19 participants would be required in each group with alpha at 0.05 and a power of 0.80. Effect sizes were calculated, where η^2^ < 0.01 indicates a small effect, η^2^ between 0.01 and 0.06 indicates a medium effect, and η^2^ between 0.06 and 0.14 indicates a large effect (Lakens, 2013). We verified the appropriateness of imputation for missing data using the missing completely at random test (Little, 1988). If <10% of the total dataset was missing and the data were missing completely at random, expectation‐maximization was therefore performed to impute the missing data.

To compare all cardiac and endothelial function measures across the three timepoints, a 2 × 3 mixed factor analysis‐of‐variance (ANOVA) model was used that included assessments of the main effects of group (TRAD and STAIR) and time [BL, 4 week, 12 week] and interaction of group by time. Pairwise comparisons post‐hoc analysis was conducted for significant interactions or main effects using Bonferroni adjustment for multiple comparisons. We used a 95% confidence interval, and statistical significance was considered at *p* < 0.05. All statistical analyses were performed using IBM SPSS Statistics for Macintosh OSX (version 20.0.0; IBM Comp., Armonk, NY, USA).

## 4 RESULTS

Eighteen participants (16M/2F) completed the exercise interventions (Table 1). Participants adhered to their respective exercise programs, TRAD (3 ± 2 day/week) and STAIR (3 ± 3 day/week), throughout the 12 week intervention (Table 2). As reported previously (Dunford et al., 2021), V̇O_2peak_ was higher after both 4 and 12 weeks of training compared to baseline (main effects), with no between‐group differences (*p *= 0.994). The CONSORT flow diagram is reported in Dunford et al. (2021). There were technical challenges with collecting echocardiographic data for two participants at two timepoints for apical rotation and one timepoint for basal rotation, which affected the calculations of left ventricular twist for those timepoints. This amounted to 1% missing data of the total dataset. Missing completely at random test revealed the data were missing completely at random (χ^2^ 16.744, *df* = 29, *p *= 0.97); therefore, we conducted expectation randomization to impute these missing values.

**TABLE 1:** Sex‐disaggregated participant characteristics

	Baseline			
	TRAD (*n* = 9)		STAIR (*n* = 9)	
	Male (*n* = 8)	Female (*n* = 1)	Male (*n* = 8)	Female (*n* = 1)
Age (yrs)	61 ± 9	52	62 ± 6	69
Height (cm)	173 ± 3	168	176 ± 6	168
Body mass (kg)	95 ± 19	99	86 ± 8	84
BMI (kg/m^2^)	29.7 ± 4.1	35	29.8 ± 3.3	30
Resting SBP (mmHg)	116 ± 18	139	113 ± 17	106
Resting DBP (mmHg)	71 ± 10	83	78 ± 7	70
Resting HR (bpm)	68 ± 10	63	74 ± 12	72
Clinical				
STEMI (*n*, %)	1 (12.5)	0 (0)	2 (25)	0 (0)
NSTEMI (*n*, %)	4 (50)	1 (100)	4 (50)	1 (100)
Angina (*n*, %)	3 (37.5)	0 (0)	2 (25)	0 (0)
PCI (*n*, %)	5 (62.5)	0 (0)	6 (75)	1 (100)
CABG (*n*, %)	3 (37.5)	1 (100)	2 (25)	0 (0)
Time since event (weeks)	8.2 ± 3.9	2	8.1 ± 5.3	13
Medications				
Beta‐blockers (*n*, %)	8 (100)	0 (0)	7 (87.5)	1 (100)
ACE inhibitors (*n*, %)	5 (62.5)	0 (0)	7 (87.5)	1 (100)
ASA (*n*, %)	8 (100)	1 (100)	8 (100)	1 (100)
Lipid lowering (*n*, %)	8 (100)	1 (100)	8 (100)	1 (100)
Metformin (*n*, %)	2 (25)	0 (0)	1 (12.5)	0 (0)
CVD risk factors				
Previous smoking history (*n*, %)	2 (25)	1 (100)	2 (25)	0 (0)
T2DM (*n*, %)	3 (37.5)	0 (0)	1 (12.5)	0 (0)
Hypertension (*n*, %)	7 (87.5)	1 (100)	5 (62.5)	1 (100)
Previous cardiac event (*n*, %)	4 (50)	1 (100)	0 (0)	0 (0)
Dyslipidemia (*n*, %)	7 (87.5)	1 (100)	6 (75)	0 (0)

Note

Data are mean ± SD.

Abbreviations: ACE, angiotensin‐converting enzyme; ASA, acetylsalicylic acid; BMI, body mass index; BP, blood pressure; CABG, coronary artery bypass graft; CVD, cardiovascular disease; HR, heart rate; NSTEMI, non‐ST‐elevation myocardial infarction; PCI, percutaneous intervention; STEMI, ST‐elevation myocardial infarction; T2DM, type 2 diabetes mellitus.

**TABLE 2:** Exercise training data for both exercise interventions

	TRAD (*n* = 9)	STAIR (*n* = 9)
Average training responses during per week (supervised; weeks 0–4)		
% Adherence	100%	100%
% Peak HR	89 ± 1	106 ± 11
% HRR	67 ± 4	99 ± 9
Peak RPE	13 ± 2	12 ± 2
Average training responses per week (unsupervised; weeks 4–12)		
% Adherence	111 ± 9%	126 ± 13%
% Peak HR	87 ± 8	96 ± 8
% HRR	77 ± 6	109 ± 7

Note

Data are mean ± SD. HR, heart rate; HRR, heart rate reserve; RPE, ratings of perceived exertion.

### 4.1 Exercise training

There were no differences in adherence regardless of exercise training group, however, the STAIR group exercised at a higher exercise intensity, measured by percent of peak heart rate and percent of heart rate reserve, during both the supervised and unsupervised exercise training (Table 2). The full details regarding the response to exercise training over 4 and 12 weeks of TRAD and STAIR are previously published in Dunford et al. (2021).

### 4.2 Effect of exercise interventions on cardiovascular function

**FIGURE 3:**
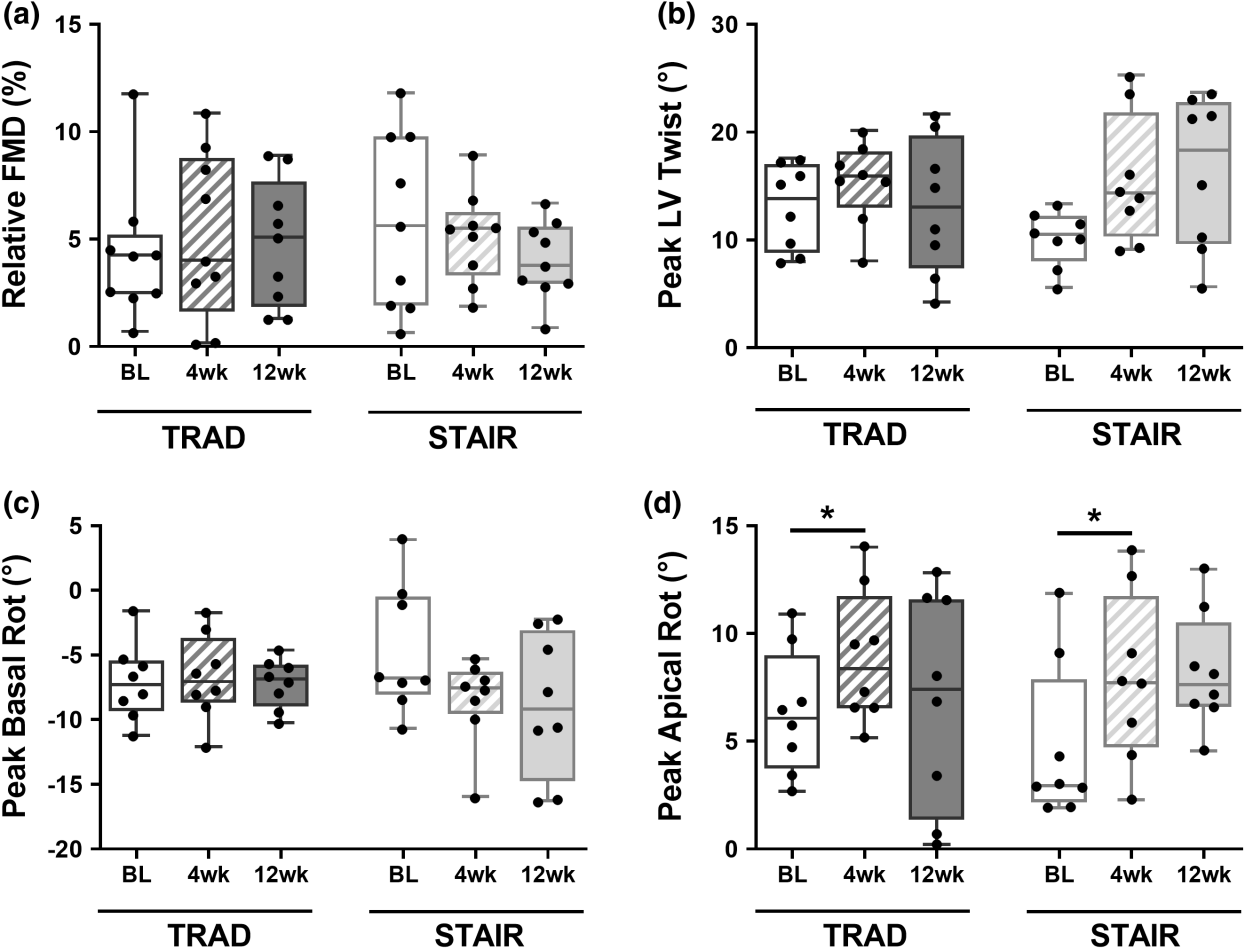
Individual values overlayed on the box and whisker format of baseline (BL), 4, and 12 week for each of the following: (a) relative flow‐mediated dilation (FMD), (b) peak left ventricular twist, (c) peak basal rotation, and (d) peak apical rotation. *Denotes *p* < 0.05

There were no group or time differences in relative FMD (%) (TRAD: BL: 4.3 ± 3.2%, 4 week: 5.12 ± 3.9%, 12 week: 4.8 ± 2.9% and STAIR: BL: 5.8 ± 4.1%, 4 week: 5.1 ± 2.1%, 12 week: 4.0 ± 1.8%, *p* (group) = 0.82, η^2^ < 0.01; *p* (time) = 0.72, η^2^ = 0.02; *p* (group × time) = 0.47, η^2^ = 0.05). There were no differences between groups for any of the clinical cardiac variables (Table 3). All additional measures of cardiac and endothelial function are displayed in Table 4. Results for relative flow‐mediated dilation, left ventricular twist, basal rotation, and apical rotation are shown in Figure 3. There were no differences in peak left ventricular twist over time nor between groups (TRAD: BL: 12.2 ± 4.6°, 4 week: 14.1 ± 5.4°, 12 week: 12.2 ± 6.7° and STAIR: BL: 11.1 ± 3.6°, 4 week: 14.4 ± 6.8°, 12 week: 15.6 ± 7.1°, *p* (group) = 0.70, η^2^ = 0.01; *p* (time) = 0.20, η^2^ = 0.10; *p* (group × time) = 0.34, η^2^ = 0.07) and peak basal rotation did not change (TRAD: BL: −7.1 ± 2.7º, 4 week: −6.3 ± 3.2º, 12 week: −6.9 ± 2.0º and STAIR: BL: −5.2 ± 5.0º, 4 week: −7.6 ± 4.0º, 12 week: −6.3 ± 3.2º, *p* (group) = 0.73, η^2^ = 0.02; *p* (time) = 0.52, η^2^ = 0.02; *p* (group × time) = 0.38, η^2^ = 0.04). Peak apical rotation increased with time (TRAD: BL: 5.6 ± 3.3º, 4 week: 8.0 ± 3.9º, 12 week: 6.2 ± 5.1º and STAIR: BL: 5.1 ± 3.6º, 4 week: 7.4 ± 3.9º, 12 week: 7.8 ± 2.8º, *p* (group) = 0.92, η^2^ < 0.001; *p* (time) = 0.03, η^2^ = 0.20; *p* (group × time) = 0.38, η^2^ = 0.06). Post‐hoc analysis of the main effect of time revealed a difference between baseline and 4 week (*p *= 0.02) and no difference between any other timepoints. There were no other differences observed for any other clinical or additional cardiovascular variables.

**TABLE 3:** Standard measures of cardiac function at baseline, 4 weeks, and 12 weeks in TRAD and STAIR groups (*n* = 9/group)

*N*, 9/group	TRAD			STAIR			*p*‐value (group)	*p*‐value (time)	*p*‐value (group × time)
	Baseline	4 week	12 week	Baseline	4 week	12 week			
LV mass (g)	258 ± 113	261 ± 108	275 ± 129	232 ± 75	256 ± 64	236 ± 49	0.57	0.69	0.58
LVM/BSA (g/m^2^)	124 ± 50	125 ± 46	133 ± 55	110 ± 36	123 ± 30	114 ± 23	0.53	0.65	0.56
EDV (ml)	156 ± 44	163 ± 65	167 ± 58	148 ± 38	147 ± 27	141 ± 27	0.42	0.85	0.37
ESV (ml)	88 ± 31	94.6 ± 43.7	101 ± 42	76 ± 33	78 ± 24	78 ± 25	0.28	0.35	0.53
SV (ml)	78 ± 16	78 ± 25	74 ± 19	68 ± 13	75 ± 15	81 ± 14	0.77	0.45	0.12
CO (l/min)	4.0 ±.8	3.9 ± 1.0	4.2 ±.6	4.2 ±.8	4.4 ±.9	4.1 ±.9	0.52	0.99	0.54
EF (%)	46 ± 10	45 ± 7	41 ± 10	52 ± 13	49 ± 7	50 ± 13	0.15	0.21	0.69
E/A ratio	1.15 ± 0.57	1.06 ± 0.38	1.18 ± 0.53	1.19 ± 0.27	1.19 ± 0.35	1.22 ± 0.32	0.65	0.76	0.88

Note

All values are expressed in mean ± SD.

Abbreviations: CO, cardiac output; E/A ratio, ratio of early passive filling to late active filling of the left ventricle; EDV, end‐diastolic volume; EF, ejection fraction using Simpson's monoplane method; ESV, end systolic volume; LV mass, left ventricular mass; LVM/BSA, left ventricular mass/body surface area; SV, stroke volume.

**TABLE 4:** Additional measures of cardiac function and brachial artery FMD at baseline, 4 weeks, and 12 weeks in TRAD and STAIR groups (*n* = 9/group)

*n* = 9/group	TRAD			STAIR			*p*‐value (group)	*p*‐value (time)	*p*‐value (group × time)
	Baseline	4 week	12 week	Baseline	4 week	12 week			
GLS (%)	−11.3 ± 3.3	−13.1 ± 2.6	−12.2 ± 2.6	−11.9 ± 3.5	−13.1 ± 2.6	−10.9 ± 2.4	0.40	0.77	0.32
GLS rate (sec^−1^)	−0.57 ± 0.020	−0.53 ± 13	−0.63 ± 0.18	−0.61 ± 0.19	−0.58 ± 0.14	−0.63 ± 0.14	0.62	0.18	0.82
MPI	0.38 ± 0.10	0.48 ± 0.13	0.44 ± 0.15	0.48 ± 0.13	0.44 ± 0.15	0.43 ± 0.13	0.70	0.72	0.21
Systolic twisting velocity (º.s^−1^)	74.7 ± 22.3	62.5 ± 22.1	58.1 ± 22.1	60.6 ± 27.0	71.0 ± 29.6	63.3 ± 26.4	0.99	0.66	0.35
Systolic basal rotation velocity (º.s^−1^)	−49.6 ± 16.1	−39.6 ± 18.6	−37.6 ± 14.1	−38.4 ± 24.3	−43.5 ± 18.8	−37.8 ± 25.6	0.69	0.61	0.48
Systolic apical rotation velocity (º.s^−1^)	34.2 ± 15.6	37.8 ± 18.1	31.7 ± 14.6	38.8 ± 12.2	37.3 ± 16.0	37.6 ± 9.8	0.51	0.77	0.71
Diastolic untwisting velocity (º.s^−1^)	−56.1 ± 15.9	−64.2 ± 22.8	−60.8 ± 23.3	−51.46 ± 19.8	−75.6 ± 29.6	−64.2 ± 31.9	0.71	0.06	0.47
Resting diameter (mm)	4.0 ± 0.6	4.1 ± 0.7	4.1 ± 0.8	4.6 ± 0.7	4.6 ± 0.6	4.6 ± 0.7	0.13	0.59	0.61
Peak RH diameter (mm)	4.1 ± 0.7	4.3 ± 0.7	4.3 ± 0.8	4.8 ± 0.8	4.8 ± 0.6	4.7 ± 0.8	0.10	0.40	0.13
Absolute FMD (mm)	0.1 ± 0.1	0.2 ± 0.2	0.2 ± 0.1	0.3 ± 0.2	0.2 ± 0.1	0.2 ± 0.1	0.28	0.58	0.13
Resting BF (ml/min)	584 ± 172	602 ± 206	601 ± 252	757 ± 229	858 ± 465	759 ± 237	0.08	0.43	0.71
Peak RH BF (ml/min)	775 ± 224	791 ± 273	824 ± 293	966 ± 261	1166 ± 540	1009 ± 300	0.10	0.39	0.14
MBV to peak (cm/s)	91.9 ± 8.3	89.0 ± 6.2	87.9 ± 5.4	86.7 ± 4.9	98.7 ± 23.2	87.5 ± 5.0	0.67	0.26	0.11
SR AUC to peak (×10^3^)	7.0 ± 5.4	6.7 ± 5.6	5.9 ± 4.4	6.6 ± 3.4	7.6 ± 5.4	8.2 ± 5.1	0.66	0.99	0.53
Time to peak (s)	43.2 ± 15.1	43.1 ± 14.0	38.2 ± 14.9	53.9 ± 27.6	48.2 ± 16.5	46.8 ± 14.3	0.14	0.57	0.88

Note

All values are expressed in mean ± SD.

Abbreviations: *, significant main effect of time, *p* ≤ 0.05; Absolute FMD, absolute flow‐mediated dilation; GLS, global longitudinal strain; MBV to peak, mean blood velocity to peak; MPI, myocardial performance index; Peak RH BF, peak reactive hyperemia blood flow; Peak RH diameter, peak reactive hyperemia diameter; Resting BF, resting blood flow; SR AUC to peak, shear rate area under the curve.

## 5 DISCUSSION

The main findings of this study are two‐fold. First, apical rotation increased following 4 weeks of both TRAD and STAIR exercise training. Second, there were no further changes in any other clinical or additional measures of cardiovascular function over time with either training program, despite the observed increases in cardiorespiratory fitness previously reported (Dunford et al., 2021).

The cross helical pattern of the left ventricular fibres from the apex to the base cause opposing rotation of the base and apical layers of the heart, which can be disrupted depending on the location of the infarct in individuals with CAD (Omar et al., 2015; Sengupta et al., 2008; Stöhr et al., 2016). In 247 people from age 18–80, of which 55 volunteers were 56–80 years old, average degrees of rotation were calculated: basal rotation of −8.2 ± 3.1º, apical rotation of 14.8 ± 7.3º, and left ventricular twist of 23 ± 8.0º (Kocabay et al., 2014). This finding supports previous research suggesting that apical rotation may be responsible for driving changes in left ventricular twist with CAD and other cardiac conditions (Kim et al., 2007; Marzolini et al., 2012; Toumanidis et al., 2013). We found a large effect size of time for changes in apical rotation, and a medium effect size of time for both left ventricular twist and basal rotation. These results for left ventricular twist and basal rotation indicate that the magnitude of the difference over time (training), despite lack of statistical significance in left ventricular twist and basal rotation in this small number of individuals. There is growing evidence that an increase in left ventricular twist is associated with exercise training; however, there is no consensus due to the small number of studies conducted and the differences in populations examined. In older patients with CAD, TRAD was previously found to result in reductions in left ventricular twist (McGregor et al., 2018), whereas in young, healthy sedentary men, a reduction in peak basal and apical rotation occurred after only 6 weeks of HIIT with no change after TRAD or in non‐exercising controls (Huang et al., 2019). In young, healthy male athletes, intensive endurance exercise training increased left ventricular twist after 3 months. However, after 39 months of exercise training, there were chronic remodeling adaptations characterized by reductions in left ventricular twist compared to the 3 month post‐training timepoint and the pre‐training baseline (Weiner et al., 2015). It seems that while a chronic reduction in left ventricular twist may indicate an improved cardiac function in young, healthy individuals, myocardial structural and functional adaptations may be dependent on both the duration of the training and the population characteristics. Despite the increased apical rotation observed after 4 weeks of exercise training in individuals with CAD, our data showed no changes in left ventricular twist. More research is required to determine the time course and direction of exercise training‐associated changes in left ventricular twist in different populations.

A meta‐analysis reported exercise training‐associated increases in left ventricular ejection fraction following at least 12 weeks of supervised continuous moderate‐intensity exercise training of 60–90 min per week (Chen et al., 2017). These changes were indicative of long‐term cardiac reverse remodeling in patients with CAD (Awadalla et al., 2017; White et al., 1987). This relatively long time‐course for exercise training associated with improvements in left ventricular function contrasts with the more rapid adverse left ventricular remodeling observed within 14 days of a myocardial event (McKay et al., 1986). The earliest documented exercise training associated myocardial function improvements have been found at 2 weeks in young, healthy males completing HIIT exercise. These improvements were measured as increases in apical rotation and left ventricular twist in parallel with increased cardiorespiratory fitness (O’Driscoll et al., 2018). Given that the time course of remodeling after a cardiac event typically is measured as an improvement in ejection fraction at 3 months, perhaps these changes in these additional measures of left ventricular function may have preceded any future changes in clinical measures of cardiac function.

While we hypothesized an improvement in myocardial performance index following exercise training, we observed no differences. Previous research suggests that individuals with CAD demonstrate improved myocardial performance index after exercise training prescribed at 90% of documented anaerobic threshold two to three times per week for 6 months, with no change in ejection fraction (Ueshima et al., 2005). Similar to our results, another study involving patients with heart failure completing either 8 weeks of home or hospital‐based exercise training found no difference in myocardial performance index, reported as Tei index (Karapolat et al., 2009). Longer training durations may be required to elicit changes in myocardial performance index. Despite these differences, training studies in this population are uncommon, and the data presented here provides additional evidence regarding myocardial performance index to the currently limited literature.

In line with our hypothesis, global longitudinal strain did not change with exercise training. These results are similar to previous work that documented no change in global longitudinal strain between groups performing aerobic interval training, aerobic training (Moholdt et al., 2009; Van De Heyning et al., 2018), or moderate‐intensity training (Maufrais et al., 2014; Van De Heyning et al., 2018). While global longitudinal strain does not seem to be affected by exercise training, this measure has been found to provide better prognostic value, compared to ejection fraction, at documenting improvements in left ventricular function (Abate et al., 2012).

Regardless of the exercise program, we observed no exercise training‐associated improvements in endothelial function (Figure 3). Despite these results, the observed differences may be clinically relevant given that a change of 1% in brachial artery FMD, has been suggested to lead to a 13% decrease in future cardiovascular event risk (Green et al., 2011). There was a small effect of group, a small effect of time, and a medium effect size of group by time for both absolute and relative FMD. Previous work has shown that endothelial function is improved after 12 weeks of supervised HIIT (Currie et al., 2013) and moderate‐intensity continuous training (Cornelissen et al., 2014) in patients with CAD. Further work by Tanaka et al. (2018) retrospectively examined brachial FMD in 60 patients with heart failure after 5 months of exercise‐based cardiac rehabilitation (2–3 exercise sessions per week for 20 min at anaerobic threshold) and found no improvement in FMD. However, patients with endothelial dysfunction, defined as FMD ≤5%, improved their exercise capacity and were independent of the change in FMD. In another study, Peller and colleagues found that participants with stable CAD completing exercise‐based cardiac rehabilitation (3–4 times per week for 40–50 min per session at a target HR zone) did not have any change in endothelial function, measured by reactive hyperemia peripheral arterial tonometry (Peller et al., 2016). Amongst these two previous studies, a common factor was improved endothelial function in those with the lowest baseline FMD values, which corroborates with other research where patients with CAD and the lowest cardiorespiratory fitness may experience the greatest gains following exercise‐based cardiac rehabilitation (Martin et al., 2013; Seals et al., 2019). In this present study, the baseline cardiorespiratory fitness levels of the sample population are higher than previous work; therefore, they did not have a similar change in relative FMD (Ramos et al., 2015). There was a moderate magnitude of difference in absolute and relative endothelial function found, depending on the and number of weeks (time) and intensity of training (group). The participants in our study may have needed additional exercise stimuli (volume, intensity, duration) during the 8 weeks of unsupervised exercise to elicit measurable cardiovascular improvements within the 12 weeks of the study.

## 6 LIMITATIONS AND FUTURE DIRECTIONS

Our study had some limitations, including that we did not reach the sample size calculated as required to detect differences in endothelial function (relative FMD). We would need to recruit an additional ten participants per group to detect a 1.7% difference in relative FMD between baseline to 12 weeks. Despite an effort to balance the recruitment both sexes, the participant group was mainly male. Unfortunately, there is a large knowledge gap regarding the sex and gender differences in cardiac rehabilitation (Ghisi et al., 2019), especially regarding cardiac remodeling and vascular function. Women may have a lower cardiorespiratory fitness when entering exercise‐based cardiac rehabilitation however the change in peak oxygen uptake is similar after 8 weeks (3 times per week) of exercise training described using an observational retrospective analysis (Witvrouwen et al., 2021). Given that exercise‐based cardiac rehabilitation is classified as Class 1A level of evidence, it would be unethical to have a non‐exercise control group or restrict their exercise to strictly the prescribed exercise protocols (Amsterdam et al., 2014; Fraker & Fihn, 2007; Smith et al., 2011). However, this lack of control is a limitation to our study design. Our recruitment strategy focused only on those patients with CAD who had already elected to participate in exercise‐based cardiac rehabilitation. Furthermore, previous research has already demonstrated declines in our primary outcome variables in a non‐exercise control group of individuals with similar CAD characteristics (McGregor et al., 2018; Wisloff et al., 2007).

## 7 CONCLUSION

While we observed increased cardiorespiratory fitness and apical left ventricular rotation in response to both exercise programs, there were no additional improvements in cardiac or endothelial function with training. These results suggest apical left ventricular rotation may indicate early cardiac adaptation to exercise training in individuals with CAD completing exercise‐based cardiac rehabilitation. Both TRAD and HIIT resulted in an increase in apical left ventricular rotation in response to exercise training, however, there is no evidence that either training program elicited any change in either clinical or additional measures of cardiovascular function in individuals with CAD.

## AUTHOR CONTRIBUTIONS

S.E. Valentino, E.C. Dunford, and M. J. MacDonald developed the concept. S.E. Valentino,, E. Lonn, M.J. Gibala, and S. M. Phillips were consulted to further enhance the study. E.C. Dunford, S.E. Valentino, E. Lonn, and J. Dubberley were involved in subject recruitment and data collection. S.E. Valentino and E.C. Dunford completed the data analysis and completed all suggested revisions from the authors. S.E. Valentino, E.C. Dunford, and M. J. MacDonald reviewed the data. S.E. Valentino wrote the first draft and developed the figures. All authors provided the feedback and direction in the revision stage and provided the feedback on the entire review and final approval for submission.
